# Single-Dose 13-Valent Conjugate Pneumococcal Vaccine in People Living With HIV – Immunological Response and Protection

**DOI:** 10.3389/fimmu.2021.791147

**Published:** 2021-12-20

**Authors:** Juliette Romaru, Mathilde Bahuaud, Gauthier Lejeune, Maxime Hentzien, Jean-Luc Berger, Ailsa Robbins, Delphine Lebrun, Yohan N’Guyen, Firouzé Bani-Sadr, Frédéric Batteux, Amélie Servettaz

**Affiliations:** ^1^ Department of Internal Medicine, Clinical Immunology and Infectious Diseases, Reims University Hospital, Reims, France; ^2^ Plateforme d’Immunomonitoring Vaccinal, Laboratory of Immunology, Cochin Hospital and University Paris-Descartes, APHP, Paris, France; ^3^ Department of Internal Medicine and Infectious Diseases, CH de Charleville-Mézières, Charleville-Mézières, France; ^4^ Laboratory of Immunology, EA7509 IRMAIC, University of Reims Champagne-Ardenne (URCA), Reims, France

**Keywords:** HIV infection, pneumococcal conjugate vaccine (PCV), immunogenicity, immunological protection, immunological response, France, cohort study

## Abstract

**Background:**

Patients living with HIV (PLHIV) are prone to invasive pneumococcal disease. The 13-valent conjugated pneumococcal vaccine (PCV13) is currently recommended for all PLHIV, followed in most guidelines by a 23-valent polysaccharide pneumococcal vaccine. Data are scarce concerning the immunological efficacy of PCV13 among PLHIV.

**Objective:**

To assess the immunological response at one month, and the immunological protection at 1-, 6-, and 12 months in PLHIV with a CD4 cell count above 200 cells/µl after a single dose of PCV13, as measured by both ELISA and opsonophagocytic assay (OPA).

**Methods:**

PLHIV with CD4 cell count >200 cells/µl were included. Specific IgG serum concentrations for eight serotypes by ELISA and seven serotypes by OPA were measured at baseline, 1-, 6-, and 12 months after the PCV13 vaccination. Global response was defined as a two-fold increase from baseline of specific IgG antibody levels (μg/ml) assayed by ELISA or as a four-fold increase in OPA titer from baseline, for at least five serotypes targeted by PCV13. Global protection was defined as an IgG-concentration ≥1 µg/ml by ELISA or as an opsonization titer ≥LLOQ by OPA for at least five tested serotypes targeted by PCV13. Factors associated with global response and global protection were assessed using logistic regression.

**Results:**

Of the 38 PLHIV included, 57.9% and 63.2% were global responders, 92.1% and 78.9% were globally protected at one month, and 64.7% and 55.9% were still protected at 12 months, by ELISA and OPA respectively. A CD4/CD8 ratio of >0.8 was significantly associated with a better global response by OPA (OR=6.11, p=0.02), and a CD4 nadir <200 was significantly associated with a poorer global response by ELISA (OR=0.22, p=0.04). A CD4 cell count nadir <200 and age over 50 years were associated with poorer global protection by OPA at M1 (OR=0.18, p=0.04) and M12 (OR= 0.15, p=0.02), respectively. Plasma HIV RNA viral load <40 copies/ml was significantly associated with a better global protection at M1 by ELISA and OPA (OR=21.33, p=0.025 and OR=8.40, p=0.04)

**Conclusion:**

Vaccination with PCV13 in these patients induced immunological response and protection at one month. At one year, more than half of patients were still immunologically protected.

## Introduction

*Streptococcus pneumoniae*, formerly known as pneumococcus, causes severe infections such as bacterial pneumonia and invasive pneumococcal diseases (IPD) including meningitis, arthritis, and bacteremia. They are responsible for a high morbidity and mortality burden. *S.pneumoniae* possess a polysaccharide (PS) pneumococcal capsule which partly explains its virulence. More than 90 different serotypes have been distinguished (1) on the basis of the capsular antigen.

People living with HIV (PLHIV) are at higher risk of invasive pneumococcal diseases (IPD) than the general population. Despite the significant risk reduction conferred by combination antiretroviral therapy (cART) (2), PLHIV are still more susceptible to IPD and have a higher risk of recurrent pneumococcal infection in the year afterward (3–5). Factors likely to explain these differences are loss of memory cells, persistent inflammation, and nasopharyngeal pneumococcal carriage (5, 6).

To prevent pneumococcal disease, most guidelines, including those in France, recommend pneumococcal vaccines in PLHIV (7–9). In Europe, two vaccine formulations are currently available: the 23-valent pneumococcal polysaccharide vaccine (PPV23) and the 13-valent pneumococcal conjugate vaccine (PCV13). The PPV23 leads to a T-independent response, which limits the magnitude of the response and duration. The PCV13 elicits a T-cell dependent immune response (10, 11), characterized by improved antibody response, induction of memory cells, and increased response duration (11).

Two techniques can be used to measure immunological response: enzyme-linked immunosorbent assay (ELISA) and opsonophagocytic assay (OPA) (12, 13). ELISA only measures antibody levels whereas the OPA can measure the functional immunogenicity of the specific serotype antibodies produced and thereby likely reflects the *in-vivo* response more accurately (13). The OPA may therefore be a better surrogate marker of immune protection in immunocompromised patients than the ELISA which may be measuring nonfunctional antibodies (13–15).

An initial PCV13 is currently recommended for all PLHIV, followed in most guidelines by a PPV23 vaccine at least 2 months later to broaden serotype coverage (7–9, 16). The clinical efficacy of this combined vaccination schedule in PLHIV is unknown (17).

The optimal timing for the initial PCV13 vaccine remains unclear, but preferentially when there is a CD4 cell count above 200 cells/µl and an undetectable viral load to strengthen immunological response.

Data are scarce concerning the immunological response to PCV13 vaccine among PLHIV, leading to a low overall pneumococcal vaccine coverage in this population (17).

The objective of the present study was therefore to assess the immunological response at one month and immunological protection at 1-, 6-, and 12 months after PCV13 administration, using both ELISA and OPA assays in a French cohort of PLHIV with a CD4 cell count above 200 cells/µl.

## Materials and Methods

### Study Design

This prospective cohort study was conducted at the Reims University Hospital from December 2013 to July 2016.

### Study Population

HIV1-infected subjects aged ≥18 years old with a CD4 T-cell count > 200 cell/µl, who had no history of anti-pneumococcal conjugate vaccination, had not received any pneumococcal polysaccharide vaccine in the last 3 years, and agreed to participate in the present study were included the day of their PCV13 vaccination. This was an exploratory observational study and recruitment was done using a pragmatic approach: all individuals fulfilling vaccination criteria and consenting to routine vaccination according to French guidelines were eligible and asked to participate in the study. The appropriate sample size could not be calculated beforehand due to the absence of previously published data for global response and protection definitions with ELISA and OPA with PCV13.

### Data Collection at Baseline and During Follow-Up

At inclusion, a blood sample was collected for all patients before vaccination (M0), then a single 0.5 ml intramuscular injection of PCV13 (Prevenar13^®^; Pfizer) was administered according to French guidelines.

The patients continued routine HIV care and were followed for up to one year without injection of further pneumococcal polysaccharide vaccine. Blood samples for immunological assessment were collected at baseline (M0) and during follow-up at 1- (M1), 6- (M6), and 12 months (M12).

Sociodemographic characteristics, clinical data, and blood test results were collected at baseline using a dedicated case report form. HIV viral load and CD4 cell count were collected at baseline, and then at 1-, 6-, and 12- months when available.

### Immunogenicity Assessment

All immunogenicity analyses were performed at the French referral laboratory for anti-pneumococcal serology and OPA (Cochin Hospital Center, Paris).

#### ELISA

IgG antibodies concentrations for eight pneumococcal serotypes (4, 6B, 9V, 14, 18C, 7F, 19F, and 23F) targeted by both PCV13 and PPSV23 and two targeted only by PPSV23 (10F and 15B) were determined using modified ELISA as previously described (18–21). Plates (Corning, Inc., Corning, NY) were coated with a serotype-specific pneumococcal PS antigen (American Type Culture Collection, Manassas, VA). Reference sera (007sp), control sera, or patient specimens were pre-absorbed with 5 µg/ml pneumococcal C-polysaccharide (Statens Serum Institut, Copenhagen, Denmark) and 10 µg/ml serotype 22F capsular polysaccharide (American Type Culture Collection). Anti-pneumococcal antibody levels were determined in each specimen by analysis of linear regression plots compared with the reference serum (007sp) (National Institute for Biological Standards and Control) (NIBSC) (21).

#### OPA

OPA was performed for seven specific serotypes (4, 6B, 9V, 14, 18C, 19F, and 23F) as previously described (18, 21, 22) to determine functional antibody responses and measured by a multiplexed opsonophagocytic killing assay (MOPA). A detailed protocol is available online at www.vaccine.uab.edu. All serum samples were incubated at 56°C for 30 min before being tested. Sera were serially diluted in round-bottom 96-well plates (Corning Inc., Corning, NY). Frozen aliquots of target pneumococci were thawed, washed twice, diluted to a bacterial density of ∼50,000 CFU/ml, and added to the plates. After 30 min of incubation at room temperature with shaking at 700 rpm, complement and HL60 cells (ATCC) that had been differentiated to phagocytes were added to each well. Plates were incubated in a tissue culture incubator (37°C, 5% CO2) with shaking at 700 rpm. After a 45-min incubation, plates were placed on ice for 20 min. Ten µl of each well were spotted onto four different Todd-Hewitt broth-yeast extract agar plates. After application of an overlay agar containing one of four antibiotics to each agar plate and overnight incubation at 37°C, the number of bacterial colonies in the agar plates was enumerated. Opsonization titers (OT) were defined as interpolated reciprocal serum dilution that killed 50% of the bacteria in the assay. The assay sensitivity is the lowest dilution of sera tested (limit of detection: LOD), which is normally 4 for undiluted sera, and is the same for each serotype. However, to quantify functional antibodies with more precision, the lower limit of quantification (LLOQ) was determined for each serotype-specific assay during assay validation. The LLOQs for each serotype were: serotype 4: 24, serotype 6B: 132, serotype 9V: 39, serotype 14: 85, serotype 18C: 47, serotype 19F: 74 and serotype 23F: 30. Titers higher than the LLOQs were considered accurate, and their values were reported. Titers below the LLOQs were set to a value of 2 (half a LOD) (18, 21, 23). Before the OPA, all samples were tested on agar for their natural bactericidal effect. A drop of serum was placed on a Todd Hewitt agar onto which we also deposited a suspension of living pneumococcus. After one night at 37°C, plates were examined for inhibition of colony growth indicating the presence of bactericidal agents in the serum. In case of bactericidal effect, the serum was not tested in OPA.

#### Definitions of Serotype and Global Response and Protection

The definitions used in our study were based on previously proposed criteria for response to pneumococcal vaccine among immunocompromised patients (5, 18, 20, 21, 24):

- Serotype specific IgG response for each serotype was defined as a two-fold increase from baseline of serotype specific IgG antibody levels (μg/ml) assayed by ELISA.
-OPA response for each serotype was defined as a four-fold increase in OPA titer from baseline
-Global responder to the vaccine by ELISA or OPA was defined as a patient who developed a response for at least five of the tested serotypes targeted by PCV13
-Protection was defined as an IgG-concentration ≥1 µg/ml by ELISA or as at least an opsonization titer ≥LLOQ by OPA for the corresponding serotype.
-Global protection by ELISA or OPA was defined by a protection for at least five of the tested serotypes targeted by PCV13

### Statistical Analysis

Qualitative values were reported as number and percentage, and compared with chi square test, or with Fisher’s exact test, as appropriate. Quantitative values were reported as mean and standard deviation (SD), and compared using the Mann-Whitney U test. Serotype-specific IgG levels (µg/ml) and OPA titers were represented as geometric mean concentrations (GMC) or titers (GMT) with their corresponding 95% confidence interval (CIs), respectively, at each time point. Correlation between values obtained by ELISA and OPA at each time point for each serotype were assessed using Spearman’s Rho. The analysis of factors associated with global response at M1 and with global protection at M0, M6, and M12 was performed using univariable logistic regression. Quantitative explicative covariates were dichotomized at the median for age, CD4 and CD8 cell count, and neutrophil count (25). Due to the insufficient number of subjects included, no multivariable logistic regression was performed. A p-value below 0.05 was considered statistically significant. All statistical analyses were performed using SAS version 9.4 (SAS Institute Inc., Cary, North Carolina, USA.

### Ethics

We received written and informed consent from every patient. This study was approved by the institutional review board of the Reims University Hospital in January 2015 and was conducted in accordance with the principles of the Declaration of Helsinki.

## Results

### Baseline Demographics

A total of 38 patients were included. Their mean age was 49.1 ± 10.8 years, and 35 (92.1%) of them were male. Baseline characteristics are detailed in **Table 1**. All patients but one were treated with combined antiretroviral therapy (cART) at the time of the PCV13 vaccination. Viral load < 40 copies/ml was present in 86.8% of patients and the mean CD4 cell count was 774.9 ± 273.8 cells cells/µl. Only one patient (taking cotrimoxazole prophylaxis) received antibiotics at baseline, and no sera induced bactericidal effect *in vitro* before OPA. Mean duration since HIV diagnosis was 15.2 ± 8.0 years. No patient had previously received the PCV7 or PCV13 vaccine, whereas 29 patients (76.3%) had previously received the PPV23 vaccine with a mean time since the last PPV23 injection of 4.8 ± 0.6 years.

**Table 1:** Baseline characteristics of 38 HIV-infected subjects with CD4 counts ≥200 cells/μl vaccinated with 13-valent pneumococcal conjugate vaccine (PCV13).

	Baseline characteristics (N=38)		Missing data
Age (mean ±sd)	49.1	±10.8	0
Male sex [n(%)]	35	(92.1)	0
Born in France [n(%)]	33	86.8	0
Body mass index (kg/m^2^)(mean ±sd)	24.5	±3.9	1
Time since HIV infection diagnosis (year) (mean ±sd)	15.2	±8.0	
Mode of HIV acquisition [n(%)]			1
MSM	28	(75.7)	
Heterosexual	6	(16.2)	
IVDU	2	(5.4)	
Other	1	(2.7)	
History of invasive pneumococcal infection [n(%)]	0	(0)	0
History of bacterial pneumonia [n(%)]	5	(13.1)	0
History of pneumococcal pneumonia [n(%)]	1	(2.6)	0
History of neoplasm [n(%)]	3	(7.9)	0
History of chronic pulmonary disease [n(%)]	1	(2.6)	0
Chronic renal insufficiency [n(%)]	2	(5.3)	0
Mean creatinine blood level (µmol/l) (mean ±sd)	78.5	±15.8	
Asplenism [n(%)]	0	(0.0)	0
CDC stage [n(%)]			0
A	24	(63.2)	
B	8	(21.0)	
C	6	(15.8)	
Viral load <40 copies/ml [n(%)]	33	(86.8)	0
Current CD4 cell count (cells/µl) (mean ±sd)	774.9	±273.8	0
CD4 cell count nadir (cells/µl) (mean ±sd)	280.1	±161.6	0
< 200 cells/µl [n(%)]	12	(31.6)	
CD8 cell count (cells/µl) (mean ±sd)	1043.2	±573.8	0
CD4/CD8 ratio (mean ±sd)	0.85	±0.4	0
Leukocyte count (G/l) (mean ±sd)	6.9	±2.2	0
Lymphocyte count (G/l) (mean ±sd)	2.5	±1.0	0
Chronic HBV infection [n(%)]	2	(5.3)	0
Chronic HCV infection [n(%)]	1	(2.6)	0
Chronic alcoholism [n(%)]*	3	(7.9)	0
Active tobacco use [n(%)]**	15	(39.5)	0
On systemic corticosteroids [n(%)]	0	(0.0)	0
Under immunosuppressive therapy [n(%)]	0	(0.0)	0
On combined antiretroviral therapy [n(%)]	37	(97.4)	0
CMV seropositivity [n(%)]	30	(78.9)	3
Prior PCV7 vaccine [n(%)]	0	(0.0)	0
Prior PPV23 vaccine [n(%)]	29	(76.3)	0
Time since last PPV23 (year) (mean ±sd)	4.8	±0.6	

Sd, standard deviation;

MSM, Men who have sex with men;

IVDU, Intravenous Drug User;

CDC, Centers for disease control;

HBV, hepatitis B infection;

HCV, hepatitis C infection;

NRTI, Nucleoside reverse transcriptase inhibitors;

NNRTI, Non-nucleoside reverse transcriptase inhibitors;

PI, Protease inhibitor;

IIN , Integrase inhibitors;

PCV7, 7-valent pneumococcal conjugate vaccine;

PPV23, 23-valent pneumococcal polysaccharide vaccine.

*Chronic alcoholism: more than 20g of pure alcohol per day.

**Active tobacco use: active smokers in the last year.

### Immunological Status at Baseline Toward Selected Pneumococcal Serotypes by ELISA and OPA in PLHIV

**Figure 1:**
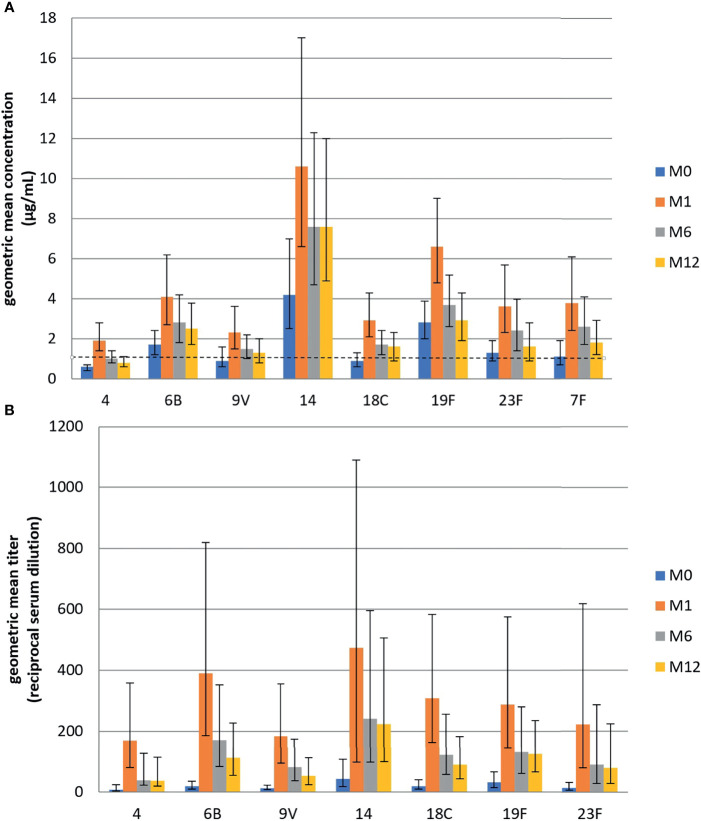
**(A)** Geometric mean concentration (GMC) of IgG antibodies toward eight pneumococcal serotypes targeted by PCV13, measured by ELISA before and one, six, and twelve months after administration of 13-valent pneumococcal conjugate vaccine among 38 HIV-infected subjects with CD4 cell count ≥200 cells/µl. **(B)** Geometric mean titers (GMT) of opsonophagocytic activity measured by OPA toward seven pneumococcal serotypes targeted by PCV13, before and one, six, and twelve months after administration of 13-valent pneumococcal conjugate vaccine at one, six, and twelve months among 38 HIV-infected subjects with CD4 cell count ≥200 cells/µl.

GMC and GMT at baseline are presented in **Table 2** and in **Figures 1A, B**. Protection rates at baseline, globally and by serotype, by ELISA and OPA, are presented in **Table 6**.

**Table 2:** Geometric mean concentrations (GMC) and geometric mean titers (GMT) to pneumococcal serotypes before and at one, six and twelve months after administration of 13-valent pneumococcal conjugate vaccine (PCV13) in 38 HIV-infected subjects with CD4 cell count ≥200 cells/µl.

Serotype	ELISA GMC								OPA GMT							
	M0		M1		M6		M12		M0		M1		M6		M12	
	GMC (µg/mL)	(95% CI)	GMC (µg/mL)	(95% CI)	GMC (µg/mL)	(95% CI)	GMC (µg/mL)	(95% CI)	GMT	(95% CI)	GMT	(95% CI)	GMT	(95% CI)	GMT	(95% CI)
4	0.6	(0.4-0.7)	1.9	(1.4-2.8)	1.0	(0.8-1.4)	0.8	(0.6-1.1)	8.3	(4.3-15.9)	169.9	(80.6-357.8)	37.6	(15.8-89.3)	36.3	(16.9-77.9)
6B	1.7	(1.2-2.4)	4.1	(2.7-6.2)	2.8	(1.8-4.2)	2.5	(1.7-3.8)	18.1	(9.1-35.8)	390.7	(186.3-819.5)	171.7	(83.6-352.5)	111.6	(54.9-226.9)
9V	1.0	(0.6-1.6)	2.3	(1.5-3.6)	1.5	(1.0- 2.2)	1.3	(0.8-2.0)	13.0	(7.3-23.1)	183.6	(94.8-355.7)	81.2	(37.6-175.0)	51.9	(23.9-112.7)
14	4.2	(2.5-7.0)	10.6	(6.6-17.0	7.6	(4.7-12.3)	7.6	(4.9-12.0)	42.8	(17.0-107.9)	473.7	(206.1-1089.1)	241.7	(97.9-596.3)	223.4	(98.8-505.4)
18C	0.9	(0.6-1.3)	3.0	(2.1-4.3)	1.7	(1.2-2.4)	1.6	(1.1-2.3)	19.6	(9.8-39.7)	309.1	(163.8-583.3)	120.6	(57.0)-255.5)	89.3	(43.8-182.2)
19F	2.8	(2.0-3.9)	6.6	(4.8-9.0)	3.7	(2.6-5.2)	2.9	(1.9-4.3)	31.1	(14.8-65.7)	287.9	(144.2-574.7)	130.7	(60.9-280.7)	124.9	(66.2-235.3)
23F	1.3	(0.9-1.9)	3.6	(2.3-5.7)	2.4	(1.4-4.0)	1.6	(0.9-2.8)	14.0	(6.1-31.8)	222.9	(80.3-618.4)	90.7	(28.8-286.0)	79.4	(28.2-223.6)
7F	1.1	(0.7-1.9)	3.8	(2.4-6.1)	2.6	(1.7-4.1)	1.8	(1.2-2.9)								
10A*	1.3	(0.7-2.3)	1.3	(0.7-2.3)	1.2	(0.7-2.3)	1.1	(0.6-2.0)								
15B*	1.8	(1.0-3.2)	2.2	(1.3-3.7)	1.8	(1.0-3.1)	1.8	(1.1-3.0)								

CI, confidence intervals.

GMC, geometric mean concentrations; GMT, geometric mean titers.

*Targeted by PPV23 but not PCV13.

At baseline, IgG antibodies GMCs were higher than the 1µg/ml threshold for all serotypes except serotypes 4, 9V, and 18 C. By contrast none of the GMTs for the seven serotypes measured by OPA were above their respective LLOQ (**Table 2**).

Baseline serotype protection in ELISA ranged from 21.0% to 86.8% of patients according to the concerned serotype. Baseline serotype protection by OPA ranged from 21.0% to 39.5% of patients according to the concerned serotype. Half of our population was considered already globally protected according to ELISA and about 15% according to OPA before PCV13 vaccination (**Table 6**).

Patients with a CD4 cell count over the population median of 750cells/µ were significantly more likely to be globally protected at baseline, when tested by ELISA (OR=4.80, p=0.03) than other PLHIV (**Table 3**). No factor was significantly associated with global protection by OPA at baseline.

**Table 3:** Factors associated with global protection at baseline by OPA and ELISA among 38 HIV-infected subjects with CD4 cell count ≥200 cells/µl.

Variable	ELISA			OPA		
	Odds ratio	Confidence interval	p	Odds ratio	Confidence interval	p
CD4 cell count > 750 cells/µl	4.80	1.20-19.13	**0.03**	2.27	0.36-14.18	0.38
CD8 cell count> 800 cells/µl	1.83	0.50-6.69	0.36	1.76	0.28-11.04	0.54
CD4/CD8 ratio > 0.8	0.43	0.12-1.59	0.21	0.50	0.08-3.13	0.46
Plasma HIV RNA viral load, <40 copies/ml	6.15	0.62-61.37	0.12	>999.99	<0.001->999.99	0.97
Neutrophil count >3500cell/µl	1.07	0.29-3.92	0.92	9.54	0.99-92.2	0.051
CDC stage C	0.12	0.01-1.15	0.07	1.08	0.10-11.32	0.95
Age > 50 years	3.67	0.95-14.09	0.06	0.88	0.15-5.05	0.89
Body mass index (kg/m^2^)	2.25	0.54-9.34	0.26	2.10	0.36-12.31	0.41
CMV seropositivity	6.91	0.68-69.86	0.10	>999.99	<0.001->999.99	0.97
Time since HIV infection diagnosis	1.44	0.39-5.28	0.58	4.41	0.46-42.13	0.20
History of bacterial pneumonia	1.25	0.18-8.50	0.82	1.40	0.13-15.26	0.78
Nadir CD4 cell count<200 cells/µL	0.45	0.11-1.80	0.26	<0.001	<0.001->999.99	0.95
Active tobacco use	0.36	0.09-1.36	0.13	0.73	0.12-4.59	0.74
Prior PPV23 vaccine	0.98	0.22-4.43	0.98	0.56	0.08-3.72	0.55

PPV23, 23-valent pneumococcal polysaccharide vaccine Globally protected was defined as a patient who developed a protection for at least five of the tested serotypes targeted by PCV13.

### One-Month Immunological Response After PCV13, Assessed by ELISA and OPA, in PLHIV

At one-month post-vaccination, we observed a 2.4- to 3.5-fold increase in GMCs compared to baseline for all serotypes contained in PCV13, and no increase in the serotypes 10A and 15B which are not present in PCV13 (**Table 2**). We found similar results by OPA, as GMTs increased 9.3–21.7-fold for all serotypes (**Table 2**). The one-month GMCs ranged from 1.3 to 10.6 µg/ml and the one-month GMTs ranged from 169.9 to 473.7.

According to the definition of immunological response for a serotype, the response rates ranged from 42.1% to 71.0% by ELISA and from 57.9 to 76.3% by OPA. Twenty-two (57.9%) and 24 (63.2%) subjects were considered to be global responders by ELISA and by OPA, respectively (**Table 4**).

**Table 4:** One-month immunological response after administration of 13-valent pneumococcal conjugate vaccine (PCV13) among 38 HIV-infected subjects with CD4 cell count ≥200 cells/µl.

Serotype	ELISA		OPA	
	N	%	N	%
4	26/38	(68.4)	27/38	(71.0)
6B	17/38	(44.7)	29/38	(76.3)
9V	19/38	(50.0)	26/38	(68.4)
14	16/38	(42.1)	22/38	(57.9)
18C	24/38	(63.2)	28/38	(73.7)
19F	18/38	(47.4)	23/38	(60.5)
23F	27/38	(71.0)	29/38	(76.3)
7F	22/38	(57.9)		
10A*	2/38	(5.3)		
15B*	3/38	(7.9)		
Global	22/38	(57.9)	24/38	(63.2)

Responses was defined as a two-fold increase of serotype specific IgG antibody levels (μ g/ml) or as a four-fold increase in OPA titer from baseline.

Global response is defined as a patient who developed a response for at least five of the tested serotypes.

*The serotypes 10A and 15B are targeted by PPV23 but not PCV13.

Factors associated with a one-month global response, by ELISA and OPA, are presented in **Table 5**. By ELISA, having had a CD4 nadir below 200 was significantly associated with a lower risk of developing a global one-month response (OR=0.22, p=0.04). By OPA, a CD4/CD8 ratio >0.8 was significantly associated with a better chance of obtaining a global one-month immunological response (OR= 6.11, p=0.019).

**Table 5:** Factors associated with global response one month after administration of 13-valent pneumococcal conjugate vaccine, by OPA and ELISA, among 38 HIV-infected subjects with CD4 cell count ≥200 cells/µl.

Variable	ELISA			OPA		
	Odds ratio	Confidence interval	p	Odds ratio	Confidence interval	p
CD4 cell count > 750 cells/µl	0.65	0.18-2.37	0.51	1.58	0.42-5.95	0.50
CD8 cell count > 800 cells/µl	1.4	0.39-5.28	0.58	0.87	0.25-3.35	0.86
CD4/CD8 ratio > 0.8	2.0	0.54-7.45	0.30	6.11	1.34-27.96	**0.02**
Plasma HIV RNA viral load, <40 copies/ml	0.30	0.03-2.98	0.30	0.38	0.039-3.84	0.41
Neutrophil count >3500cell/µl	0.89	0.24-3.28	0.86	0.60	0.16-2.28	0.45
CDC stage C	0.68	0.12-3.93	0.67	1.2	0.19-7.57	0.85
Age > 50 years	0.31	0.08-1.22	0.09	0.29	0.07-1.18	0.08
Body mass index (kg/m^2^)	2.25	0.54-9.34	0.26	2.82	0.62-12.89	0.18
CMV seropositivity	2.25	0.33-15.54	0.41	3.00	0.43-20.94	0.27
Time since HIV infection diagnosis	0.45	0.12-1.75	0.25	0.66	0.17-2.55	0.54
History of bacterial pneumonia	1.10	0.16-7.5	0.92	2.60	0.26-25.92	0.41
CD4 cell count nadir <200cells/µl	0.22	0.05-0.96	**0.04**	0.26	0.06-1.11	0.07
Active tobacco use	1.15	0.31-4.33	0.83	0.80	0.21-3.06	0.74
Prior PPV23 vaccine	0.61	0.13-2.95	0.54	1.52	0.33-6.96	0.59

PPV23, 23-valent pneumococcal polysaccharide vaccine.

Definition of a global responder: patient who developed a response for at least five of the tested serotypes targeted by PCV13.

Bold values indicate p < 0.05.

### One-Month, 6-Month, and 12-Month Immunological Protection After PCV13, Analyzed by ELISA and OPA, in PLHIV

At one month, the protection rate for each serotype included in PCV13 ranged from 71% to 97.4% by ELISA and from 76.3% to 86.8% by OPA (**Table 6**). Thirty-five (92.1%) and thirty (78.9%) patients were globally protected according to ELISA and OPA measures, respectively.

**Table 6:** Proportion of protected patients defined as patients who had a level ≥ 1µg/ml of Streptococcus pneumoniae polysaccharide-specific IgG or as at least an opsonization titer ≥LLOQ by OPA at baseline, one, six and twelve months after administration of 13-valent pneumococcal conjugate vaccine among 38 HIV-infected subjects with CD4 cell count ≥200 cells/µl.

Serotype	ELISA								OPA							
	M0		M1		M6		M12		M0		M1		M6		M12	
	N	%	N	%	N	%	N	%	N	%	N	%	N	%	N	%
4	8/38	21.0	27/38	71.0	16/36	44.4	14/34	41.2	10/38	26.3	32/38	84.2	21/35	60.0	22/34	64.7
6B	28/38	73.7	34/38	89.5	27/36	75.0	26/34	76.8	8/38	21.0	31/38	81.6	21/35	60.0	21/34	61.8
9V	17/38	44.7	2638	68.4	22/36	61.1	16/34	46.1	10/38	26.3	31/38	81.6	23/35	65.7	21/34	61.8
14	32/38	84.2	37/38	97.4	34/36	94.4	34/34	100.0	15/38	39.47	30/38	78.9	23/35	65.7	24/34	70.6
18C	20/38	52.6	33/38	86.8	26/36	72.2	22/34	64.7	14/38	36.8	33/38	86.8	25/35	71.4	23/64	67.6
19F	33/38	86.8	37/38	97.4	33/36	91.7	28/34	82.3	14/38	38.8	31/38	81.6	22/35	62.9	22/34	64.7
23F	21/38	55.3	34/38	89.5	28/36	77.8	21/34	61.8	9/38	23.7	29/38	76.3	23/35	65.7	22/34	64.7
7F	16/38	42.1	30/38	78.9	24/34	70.6	24/34	70.6								
10A*	18/38	47.4	18/38	47.4	15/34	44.1	16/34	47.1								
15B*	25/38	65.8	26/38	68.4	21/34	61.8	20/34	58.8								
Global	21/38	55.3	35/38	92.1	26/36	72.2	22/34	64.7	6/38	15.8	30/38	78.9	21/35	60.0	19/34	55.9

Protection was defined as an IgG-concentration ≥1 µg/mL by ELISA or as at least an opsonization titer ≥LLOQ by MOPA for the serotype. The LLOQs (lower limit of quantification) for each serotype were: serotype 4: 24, serotype 6B: 132, serotype 9V: 39, serotype 14: 85, serotype 18C: 47, serotype 19F: 74 and serotype 23F: 30.

Globally protected was defined as a patient who developed a protection for at least five of the tested serotypes targeted by PCV13.

*The serotypes 10A and 15B are targeted by PPV23 but not PCV13.

GMCs at 6 and 12 months remained higher than baseline for all serotypes contained in the PCV13 but decreased compared to M1 (**Figure 1A**). Only serotype 4 had GMC < 1µg/ml in ELISA at 12 months. By OPA, GMT also decreased but still exceeded the protective threshold for all serotypes at M12.

Consequently, at six months, the protection rate for each serotype included in PCV13 ranged from 44.4% to 91.7% by ELISA and 60% to 71.4% by OPA. Twenty-six (72.2%) and twenty-one (60.0%) patients were globally protected by ELISA and by OPA, respectively (**Table 6**).

At 12 months, the protection rate for each serotype included in PCV13 ranged from 41.2% to 100.0% by ELISA and 61.8% to 70.6% by OPA. Twenty-two (64.7%) and nineteen (55.9%) patients were globally protected by ELISA and by OPA, respectively (**Table 6**).

No patient developed IPD during the study and follow-up after a mean follow-up of 6.8 years.

### Factors Associated With 1-Month and 12-Months Global Protection After PCV13, Analyzed by ELISA and OPA, in PLHIV

The factors associated with one-month and twelve-month global protection, by ELISA and OPA, are presented in **Tables 7** and **8**, respectively.

**Table 7:** Factors associated with global protection one month after administration of 13-valent pneumococcal conjugate vaccine, by OPA and ELISA, among 38 HIV-infected subjects with CD4 cell count ≥200 cells/µl.

Variable	ELISA			OPA		
	Odds ratio	Confidence interval	p	Odds ratio	Confidence interval	p
CD4 cell count > 750 cells/µl (median)	>999.99	<0.001->999.99	0.95	3.92	0.68-22.70	0.13
CD8 cell count > 800 cells/µl	0.59	0.05-7.17	0.68	0.69	0.14-3.40	0.64
CD4/CD8 ratio > 0.8	>999.99	<0.001->999.99	0.95	3.43	0.59-19.80	0.17
Plasma HIV RNA viral load, <40 copies/ml	21.33	1.47-310.01	**0.025**	8.40	1.11-63.73	**0.04**
Neutrophil count >3500cell/µl	1.50	0.12-18.12	0.75	2.62	0.45-15.16	0.28
CDC stage C	0.33	0.025-4.40	0.40	0.46	0.07-3.13	0.43
Age > 50 years	0.53	0.04-6.39	0.62	0.60	0.12-2.97	0.53
Body mass index (kg/m^2^)	1.09	0.09-13.30	0.95	0.88	0.17-4.45	0.87
CMV seropositivity	3.5	0.25-48.03	0.35	0.82	0.08-8.60	0.87
Time since HIV infection diagnosis	0.67	0.05-8.06	0.75	0.78	0.16-3.90	0.77
History of bacterial pneumonia	0.26	0.02-3.53	0.31	0.33	0.04-2.45	0.28
Nadir CD4 count cell/µL<200 cells/µl	0.20	0.02-2.46	0.21	0.18	0.03-0.96	**0.04**
Active tobacco use	1.33	0.11-16.14	0.82	0.58	0.12-2.79	0.50
Prior PPV23 vaccine	<0.001	<0.001->999.99	0.95	1.09	0.18-6.69	0.92

PPV23, 23-valent pneumococcal polysaccharide vaccine.

Globally protected was defined as a patient who developed a protection for at least five of the tested serotypes targeted by PCV13.

Bold values indicate p < 0.05.

**Table 8:** Factors associated with global protection twelve months after administration of 13-valent pneumococcal conjugate vaccine, by Elisa and OPA, among 38 HIV-infected subjects with CD4 cell count ≥200 cells/µl.

Variable	ELISA			OPA		
	Odds ratio	Confidence interval	p	Odds ratio	Confidence interval	p
CD4 cell count > 750 cells/µl	4.29	0.96-19.18	0.06	1.96	0.49-7.77	0.34
CD8 cell count > 800 cells/µl	0.86	0.21-3.55	0.83	1.20	0.31-4.70	0.79
CD4/CD8 ratio > 0.8	1.68	0.40-6.96	0.47	3.43	0.83-14.21	0.09
Plasma HIV RNA viral load, <40 copies/ml	7.00	0.64-76.7	0.11	4.50	0.42-48.53	0.21
Neutrophil count >3500cell/µl	0.57	0.14-2.38	0.44	1.09	0.27-4.32	0.90
CDC stage C	0.47	0.08-2.83	0.41	0.75	0.13-4.39	0.75
Age > 50 years	1.44	0.35-5.95	0.61	0.15	0.03-0.70	**0.02**
Body mass index (kg/m^2^)	4.54	0.79-25.97	0.09	0.75	0.18-3.11	0.69
CMV seropositivity	1.33	0.19-9.46	0.77	0.27	0.03-2.73	0.27
Time since HIV infection	0.60	0.14-2.59	0.49	0.91	0.23-3.63	0.90
History of bacterial pneumonia	0.30	0.04-2.12	0.23	0.47	0.07-3.26	0.44
Nadir CD4 cell count <200 cells/µl	0.52	0.12-2.31	0.39	0.30	0.07-1.36	0.12
Active tobacco use	0.27	0.06-1.18	0.08	0.87	0.22-3.52	0.85
Prior PPV23 vaccine	0.68	0.11-4.18	0.68	0.43	0.07-2.62	0.36

PPV23, 23-valent pneumococcal polysaccharide vaccine.

Globally protected was defined as a patient who developed a protection for at least five of the tested serotypes targeted by PCV13.

Bold values indicate p < 0.05.

Patients who were global responders at one month were not significantly more likely to obtain global immunological protection at 12 months whether assessed by ELISA or OPA (p=0.28 and p=0.27, respectively).

One month after vaccination, PLHIV with a CD4 cell count nadir below 200 cells/µl were significantly less globally protected (OR=0.18, p=0.04) by OPA. PLHIV with a plasma HIV RNA viral load <40 copies/ml were significantly more frequently globally protected (OR=21.33, p=0.025 and OR=8.40, p=0.04) by ELISA and OPA, respectively.

Twelve months after vaccination, patients aged over 50 were less likely to still be protected according to OPA results (OR=0.15, p=0.02). We found no factors significantly associated with global protection at M12 by ELISA.

### Correlation Between ELISA and OPA Methods at Baseline, M1, M6, and M12, in PLHIV

Given that two techniques were systematically used to assess anti-pneumococcal immunological response and protection in our population, we analyzed the correlation between the results obtained from both techniques.

There was a significant positive correlation between values obtained for all assessed serotypes by ELISA and OPA (moderate correlation for all serotypes at each timepoint except for serotype 14 which had a strong correlation, **Table 9**).

**Table 9:** Spearman’s correlation coefficients between Elisa and OPA values at baseline, 1-, 6- and 12 month and for each serotype among 38 HIV-infected subjects with CD4 cell count ≥200 cells/µl who received the PCV13 vaccine.

Serotype	M0		M1		M6		M12	
	Rho	p	Rho	p	Rho	p	Rho	p
4	0.56	0.0003	0.46	0.003	0.54	0.0008	0.36	0.04
6B	0.54	0.0005	0.42	0.009	0.42	0.01	0.42	0.01
9V	0.44	0.006	0.38	0.02	0.50	0.002	0.48	0.004
14	0.71	<0.0001	0.72	<0.0001	0.80	<0.0001	0.81	<0.0001
18C	0.52	0.0009	0.45	0.005	0.41	0.01	0.39	0.02
19F	0.39	0.02	0.33	0.04	0.48	0.004	0.63	<0.0001
23F	0.46	0.004	0.65	<0.0001	0.44	0.007	0.53	0.001

Correlation was assessed using Spearman’s correlation coefficient [0.10-0.39: weak correlation ; 0.40-0.69: moderate correlation ; 0.70 – 0.89: strong correlation ; 0,90 – 1.00: very strong correlation (26)].

A strong correlation was found between global responses assessed by ELISA and OPA (Rho=0.67, p<0.0001). Correlations were found between OPA and ELISA for immunological responses to serotypes 4 (p=0.0002), 9V (p=0.04), 14 (p<0.0001), 18C (p<0.0001), and 19F (p=0.005).

There was a weak correlation of borderline significance between ELISA and OPA for global protection at M12 (Rho=0.33, p=0.053). For the serotype tested and methods, we found a significant serotype correlation between ELISA and OPA for only three serotypes [4 (p=0.03), 19F (p=0.006), and 23F (p=0.01)].

## Discussion

In our study, we evaluated the PCV13 immunological response and the global protection before and up to one year after a single dose of vaccination in PLHIV by combining two methods (ELISA and OPA). To the best of our knowledge, this study is the first to evaluate qualitatively (immunological response and protection) and quantitatively anti-pneumococcal humoral immunity after a single dose of PCV13 over a one-year period and its associated factors in PLHIV. These data may help clinicians improve PCV13 vaccine strategy and monitoring.

Although immunogenicity studies of PCV7 in HIV-infected adults have already been published (27–30), PCV13 vaccination-related questions in PLHIV (5, 17, 24, 31, 32) and immune response rate after a single dose of PCV13 have rarely been described. Most of the published studies concern a vaccination regimen of two doses of this conjugate vaccine whereas current guidelines for PLVIH recommend a single dose of PCV13 followed by PPV23 after at least 2 months, and even after a minimum of 1 year (8, 9).

We have observed in our study that most virologically suppressed and immunologically controlled PLHIV achieve adequate global immunological response and protection one month after a single PCV13 vaccination. According to ELISA and OPA measures, 57.9% and 63.2% were global responders and 92.1% and 78.9% were globally protected, respectively. Twelve months later, and without PPV23 subsequent vaccination, 64.7% and 55.9% of them were still globally protected against the tested serotypes, by ELISA and by OPA, respectively.

Only three studies have previously investigated vaccine response after a single dose of PCV13 in this population, either by ELISA or OPA, and the majority only analyzed two serotypes (5, 31, 32). One study was performed in PLHIV stratified by CD4 T-cell count (less or more than 350 cells cells/µl), and reported serotype response rates using the same definition at one month assessed by OPA between 60.6% and 73.5% for the serotype 6B and between 60.6% and 76.5% for the serotype 18C (5). We found comparable results by OPA analysis for these two serotypes. In a previous study using ELISA and OPA, the mean immunological response for the PCV13 vaccine was observed after one single dose of PCV13 for all but one (serotype 5 by ELISA and serotype 14 by OPA) of the 13 serotypes included in the PCV13 (32). Furthermore, the 1µg/ml threshold was achieved for all serotypes but serotype 3. However, response rates were not provided. The increase in GMC and GMT between baseline and M1 were comparable with our study (for GMC: 2.44 to 5.40-fold versus 2.4 to 3.45-fold in our study respectively, and for GMT: 9.6 to 22.7-fold versus 9.3 to 21.7-fold in our study) (32). Similar results were reported in a third study stratifying PLHIV by CD4 cell count > or <400 cells/µl, and analyzing immune response against the serotype 3 and 14 by ELISA (31).

Although disease prevention represents a better criterion than vaccine immune response for prophylactic vaccines like PCV13, we used pre-existing immunological criteria to define vaccine-induced response and protection against pneumococcal infection (18, 20, 21).

In our study, we found that IgG antibody GMCs to six serotypes (6B, 9, 14, 19F, 23F, and 7F) were already greater than or equal to 1µg/ml before the PCV13 whereas GMT were never above the LLOQ for the seven serotypes tested. These results are consistent with those reported in a previous study for several serotypes (32). Moreover, half of our population was considered as already globally protected according to ELISA and only about 15% before PCV13 vaccination according to OPA, probably due to the presence of non-functional antibodies. This has already been observed in other studies (21). The patients with CD4 cell counts greater than 750 cells/µl were significantly more frequently protected at baseline according to ELISA analysis. Interestingly, we found no association with a previous PPV23 injection although most patients had already received one.

One month after PCV13 vaccination, we found that thirty-five (92.1%) and thirty (78.9%) patients were globally protected according to ELISA and OPA measures, respectively. Only three studies have assessed vaccinal protection after a dose of PCV13 either in ELISA or OPA or both at one month after vaccination (5, 31, 32). Moreover, none of these studies reported global protection rates induced by PCV13 since their designs were different from our study. IgG levels targeting identical serotypes were comparable between our study and theirs. In a study, GMCs were similar with our study at one month and were above the 1µg/ml threshold for all but one of 13 serotypes (serotype 5) (32). At one month, in this same study, OPA GMT was higher for serotypes 4, 6B, 9V, 14, and 18C but lower for serotypes 19F and 23F than in our study (32). In another study concerning only two serotypes (3 and 14), all patients were protected for serotype 14 one month after PCV13 vaccine but with a lower increase than in our study (31). In our study, at 1 month, the protection rate for each serotype ranged from 76.3% to 86.8% by OPA and 78.9% were globally protected. In a previous study, one-month immunological protection rate by OPA varied from 87.9% to 100% for serotype 6B and from 75.8% to 94.1% for serotype 18C according to the CD4 cell count (5).

The durability of protection after PCV13 is another crucial point to further adapt vaccination strategy, and data concerning long-term protection after PCV13 in PLHIV are rare. At twelve months after PCV13 vaccine, we observed a decrease in the immunological global protection rate during follow-up and only 64.7% and 55.9% of the patients were still globally protected twelve months after their PCV13 vaccination, by ELISA and by OPA, respectively. To our knowledge, no such result has been published to date. Only one study assessed GMC one year after PCV13 vaccination for two serotypes (3 and 14), and for serotype 14, which is known as an immunogenic serotype (33). We found higher antibody concentrations at M12 (2.95 µg/ml vs. 7.6 µg/ml). Indeed, in our study, all patients were considered protected at M12 by ELISA for this serotype (70.6% by OPA). There was also a progressive decrease in antibody concentration and opsonophagocytic titers over time for all serotypes.

There is also few one-year data with ELISA and OPA in patients with primary immune deficiency (21), multiple myeloma (18), or in elderly (>65 years old) patients (34), which all observed a decrease of GMCs and GMTs over time. There is no data on the general population. In contrast to the CAPITA trial (34), which included patients over 65, we observed lower GMC values at M12 for all tested serotypes except for serotype 14 and 19F which were similar. By OPA, we observed lower GMT values at 12 months for all tested serotypes except for serotype 19F and 23F which were similar. However, no direct comparison was made.

We found a moderate correlation between antibody levels and opsonophagocytic titers at each time point for all serotypes, but a weak non-significant correlation for global protection at M12. Although part of this lack of association may be related to insufficient statistical power, and to the definitions of protection for both techniques (35, 36), these discrepancies may result from chronic viral infection-induced inflammation leading to the production of non-functional antibodies in PLHIV (37). Nevertheless, this poor correlation between ELISA and OPA results was reported in other studies in HIV-negative populations and may be related to functional variabilities between the antibodies produced. Comparison of affinity and/or avidity of anti-pneumococcal antibodies at different time points with higher and lower OPA titered sera would be a useful addition in the future to shed light on the discordance between ELISA and OPA results at different time points before and after vaccination. Consequently, in this population, as reported in elderly or in immunocompromised populations, GMC may represent insufficient surrogate markers to evaluate anti-pneumococcal immune protection (13, 37, 38). At baseline, very few patients (15%) were considered as globally protected when assessed by OPA before vaccination whereas most patients had antibody levels above 1 µg/l before vaccination by ELISA. Interestingly, we showed that one month after PCV13 vaccination, the GMC increased 2.4 to 3.45-fold from baseline whereas GMT increase was higher, from 9.26 to 21.7-fold from baseline. Thus, PCV13 elicits the production of functional antibodies in this population and improves their opsonophagocytic ability. We found a significant correlation between ELISA and OPA for the global response with Rho=0.67 (p<0.0001), underlining once again the ability of the vaccine to stimulate the production of new functional antibodies by B cells.

Few articles have investigated the factors associated with PCV13 vaccine response. We have found that a CD4/CD8 ratio > 0.8 was significantly associated with a better global response by OPA and that a CD4 nadir below 200 was significantly associated with a lower risk of global response by ELISA.

In previous studies, CD4 cell count seemed to represent the main factor associated with vaccine response, particularly with PPV23. Nadir CD4 count has previously been identified as a marker of poorer response (5, 37, 39–42). CD4 cell count nadir strongly correlates with a lower level of memory B-cells despite CD4 cell count recovery. This could limit the physiological maintenance of the B-cell compartment, thereby limiting vaccine-induced response and protection (43). In a recent study, combination antiretroviral therapy at vaccination and virological control were correlated with a better response to PPV23 vaccination (42). The CD4/CD8 ratio correlates with chronic inflammation and immune senescence, even in virologically suppressed and immunologically controlled PLHIV and has been associated with poor hepatitis A and hepatitis B vaccine responses (44–46). This parameter also seems interesting for clinicians in relation to PCV13 vaccination, because subjects nowadays are mostly virologically suppressed patients, receiving HAART and with CD4 cell count > 200 cells/µl, even > 500 cells/µl. One study suggested that the number or percentage of CD4 T lymphocytes co-expressing CD28 could predict responses to vaccination in PLHIV. This marker could be tested in future studies on pneumococcal vaccine responses (47).

In regard to factors associated with protection, we observed that people with a nadir CD4 cell count greater than 200 cells/µl and plasma HIV RNA viral load <40 copies/ml at baseline were significantly more frequently globally protected one month after vaccination. In line with these results, Song et al. reported that the protection of PCV13 was significantly inferior among HIV-infected patients with CD4 T-cell count < 350 cells/μL compared to those with higher CD4 T-cell counts (5). Being a global responder at M1 was not significantly predictive of being globally protected at M12. PLHIV over 50 were less frequently globally protected at M12, which may be explained by immunosenescence promoted by aging and HIV. For HIV viral load, this has already been observed with antibody response or antibody specificity for various vaccines, including pneumococcal vaccines (42, 48, 49), but there is no data to our knowledge for the association with protection with PCV13. This is consistent with most guidelines recommending vaccinating PLHIV who have a HIV viral load < 50 copies/ml (50).

The strength of our study lies in the fact that it is the first to study the immunological efficacy of PCV13 up to one year in PLHIV, using ELISA and OPA with many serotypes. This allowed the assessment of global response and global protection and their associated factors in a population of immunologically controlled patients, which is the main type of population observed in developed countries today.

Our study also has also several limitations. The size of the sample limits the power to assess factors associated with immunological response and protection. The factors associated with global response or protection should be verified in other studies. The overrepresentation of PLVIH born in France may have introduced a selection bias, considering the potential variability of immune responses among PLVIH of different ancestry. Furthermore, all except three of the patients were men. In a previous study in adults aged over 50 years, men were found to have consistently higher levels of serotype-specific IgG than women in a study on PCV7 and/or PP23 responses. Nevertheless, the effect of sex on humoral responses to vaccines differs between different studies and the vaccines studied (51, 52). It should also be noted that the thresholds used in the present study and in others have not been validated in clinical studies focusing on pneumococcal pneumonia or invasive pneumococcal disease and with PLVIH.

In conclusion, our study demonstrated that a single PCV13 vaccination in a population of PLHIV (mostly immuno-virologically controlled) led to immunological response and protection at one month for most patients, especially those with a CD4 nadir greater than 200 cells/µl and a CD4/CD8 ratio greater than 0.8. In most patients PCV13 improved the opsonophagocytic activity of anti-pneumococcal antibodies. After six and twelve months without a boost vaccination by PPV23, the percentage of protected patients decreased but more than half of patients were still immunologically globally protected at one year. Considering discrepancies between ELISA and OPA results, OPA seems more appropriate in a research context to assess immunological protection in this population, using published thresholds.

## Data Availability Statement

The raw data supporting the conclusions of this article will be made available by the authors, without undue reservation.

## Ethics Statement

The studies involving human participants were reviewed and approved by Institutional review board of the Reims University Hospital. Written informed consent for participation was not required for this study in accordance with the national legislation and the institutional requirements.

## Author Contributions

AS, MH, JR, and AR designed the study. J-LB, DL, FB-S, and YN’G took care of patients. JR and GL collected the data. MB performed ELISA and OPA analyses. MH performed the statistical analysis. JR, MH, and AS drafted the manuscript. AS supervised the study conduction. All authors contributed to data interpretation and analysis and revised the manuscript.

## Conflict of Interest

The authors declare that the research was conducted in the absence of any commercial or financial relationships that could be construed as a potential conflict of interest.

## Publisher’s Note

All claims expressed in this article are solely those of the authors and do not necessarily represent those of their affiliated organizations, or those of the publisher, the editors and the reviewers. Any product that may be evaluated in this article, or claim that may be made by its manufacturer, is not guaranteed or endorsed by the publisher.
